# Effects of Oxidant-Antioxidant and Vitamin D Levels on Clinical and Laboratory Data in Children With Fatty Liver Disease

**DOI:** 10.7759/cureus.11849

**Published:** 2020-12-02

**Authors:** Sukru Gungor, Ahmet Alpay Köylü, Suadiye Saglam, Salim Neselioglu, Özcan Erel, Can Acıpayam

**Affiliations:** 1 Pediatric Gastroenterology, Kahramanmaras Sutcu Imam University Medical Faculty, Kahramanmaraş, TUR; 2 Biochemistry, İzmir Katip Çelebi University Atatürk Training and Research Hospital, Izmir, TUR; 3 Biochemistry, Yildirim Beyazit University, Ankara, TUR; 4 Pediatric Hematology and Oncology, Kahramanmaras Sutcu Imam University Medical Faculty, Kahramanmaraş, TUR

**Keywords:** vitamin d deficiency, child and adolescent, hepatosteatosis, oxidant-antioxidant

## Abstract

Background and Aims

Fatty liver increases oxidative stress and may trigger antioxidant mechanisms. We aimed to compare the levels of vitamin D, which has antioxidant properties, as well as total oxidant status (TOS), total antioxidant status (TAS), and catalase between patients with nonalcoholic fatty liver (NAFL) and the control group.

Methods

We compared vitamin D, TOS, TAS, catalase levels, and other biochemical parameters between pediatric patients with ultrasonographically detected NAFL and an age-matched healthy control group.

Results

NAFL patients had a significantly lower vitamin D level (p < 0.001). The patient group also had significantly greater height, weight, body mass index (BMI) Z score, parathyroid hormone, triglyceride, glucose, antioxidant (TAS and catalase), and TOS levels compared to the controls (p ≥ 0.001). There was no significant difference between the obese and non-obese NAFL patients with respect to TAS, TOS, catalase levels, and other biochemical parameters (p < 0.05). There was a positive correlation between height, weight, BMI Z score, and hepatosteatosis grade, and TAS, TOS, and catalase levels, and a negative correlation with vitamin D level. We also found a negative correlation between vitamin D level and TOS and catalase level.

Conclusions

Our study revealed lower levels of vitamin D and higher levels of oxidant-antioxidants including TOS, TAS, and catalase in patients with NAFL.

## Introduction

Nonalcoholic fatty liver disease (NAFLD) is one of the common chronic liver disorders in children. It is characterized by elevated biochemical markers due to injury occurring secondary to hepatic fat accumulation. Although it has been related to obesity, dyslipidemia, inflammation, and insulin resistance, its etiopathogenesis has not been fully understood [1,2]. Vitamin D is a fat-soluble vitamin. It not only helps to regulate inflammation and oxidative stress in a variety of tissues but it also regulates lipid and glucose metabolism in the liver [3,4]. Patients with hepatosteatosis show an increased level of oxidative stress and impaired antioxidant defense mechanisms [5]. Roth et al. reported that vitamin D deficiency in rats led to the development and progression of nonalcoholic steatohepatitis (NASH) by augmenting the expression of genes involved in oxidative stress and inflammatory pathways [6]. Nobili et al. reported that vitamin D lowered alanine aminotransferase (ALT) level and, at the same time, improved histopathological hepatosteatosis components other than fibrosis in patients with NASH [7]. Furthermore, Weisberg et al. [8] empathized that proinflammatory molecules released from the fat tissue, which play a role in obesity-related complications, may alter metabolic and endocrinological functions by inducing free radical formation [9]. The resulting reactive oxygen radicals and nitrogen species (ROS and RNS) may affect macromolecules such as lipids, carbohydrates, proteins, and nucleic acids, causing oxidative injury [10]. Antioxidants take part in the prevention of cellular injury by lowering the number of free radicals [11]. As far as we know, no study has yet investigated levels of antioxidants in pediatric nonalcoholic fatty liver (NAFL) patients. Therefore, we aimed to compare admission vitamin D and antioxidant levels between pediatric patients with ultrasonographically detected fatty liver and the control group in order to contribute to the development of early treatment regimens.

## Materials and methods

Pediatric patients aged 1-17 years diagnosed with hepatosteatosis by ultrasonography between December 2018 and December 2019 were included in the study. Patients with fatty liver (NAFL) but normal transaminase levels were grouped as nonalcoholic hepatosteatosis (NAS), and patients with fatty liver and elevated transaminase levels unexplained by other causes were grouped as possible NASH.

This study was conducted in compliance with the Helsinki Declaration. Informed consent was obtained from the patients’ families before study participation.

Control group

Healthy children of similar age without obesity or ultrasonographically diagnosed hepatosteatosis were enrolled as the control group.

Exclusion criteria

Patients with morbid obesity, splenomegaly, chronic liver disease, diabetes, renal failure, heart failure, chronic viral hepatitis, Wilson disease, autoimmune hepatitis, α1 antitrypsin deficiency, cystic fibrosis, coeliac disease, active infection, and a history of medication use in the last month were excluded.

Anthropometric measurements

Height measurements of children participating in the study were performed with patients having their shoes and socks removed, using an infantometer for children younger than two years and a vertical portable stadiometer calibrated to the nearest millimeter for children older than two years. Weight measurements were performed with patients wearing light clothing, using a precise digital electronic scale. Age- and sex-adjusted weight Z score, height Z score, height-weight Z score, and body mass index (BMI) Z score were calculated using the World Health Organization (WHO) data.

According to the Endocrine Society Clinical Practice Guidelines, the patients were categorized as overweight when they had a BMI between 85th and 95th percentile of age and sex-adjusted values:

1. Class 1 obese when they had a BMI equal to or greater than 95th percentile

2. Class 2 obese when they had a BMI between 95th and 120th percentile

3. Class 3 extremely obese when they had a BMI equal to or greater than 120 percentiles [12,13].

Preparation of samples

Blood samples were taken from the patient and control groups between 8:00 and 10:00 in the morning following a 10- to 12-hour fasting period. Immediately after blood clotting, the samples were centrifuged at 2,500 rpm for 10 minutes to separate sera. The sera were then immediately sent for analysis of glucose, aspartate aminotransferase (AST), ALT, alkaline phosphatase (ALP), gamma-glutamyl transferase (GGT), total bilirubin, (T.bil), direct bilirubin (D.bil), calcium (Ca), phosphorus (P), parathyroid hormone (PTH), triglyceride (TG), and cholesterol levels with Cobas c501 automatic analyzer (Roche, Basel, Switzerland) using routine methods; vitamin D levels were studied in Advia Centaur® XP hormone device (Siemens Healthcare GmbH, Erlangen, Germany). Sera were preserved at -80°C until the day of biochemical analysis of catalase (CAT), total antioxidant status (TAS), and total oxidant status (TOS) levels.

Ultrasonographic definition and grades of hepatosteatosis

The grades of hepatosteatosis are as follows [14]:

1. Grade 0: Absent steatosis.

2. Grade 1: There is a slight fat infiltration in the liver parenchyma. Parenchymal echogenicity slightly increases compared to kidney. Intrahepatic vessels and diaphragm are visualized.

3. Grade 2: There is a slight impairment of the echogenicity of the liver parenchyma, visualization of the diaphragm and intrahepatic vessels. There is increased fat in the liver parenchyma.

4. Grade 3: There is moderate-to-severe fatty liver change in the liver parenchyma. Liver parenchymal echogenicity is markedly increased. The borders of the diaphragm and intrahepatic vessels are weak or not visible.

Study methods

Catalase Level

CAT activity was quantified by Goth’s method [15]. The sample (0.2 mi), 60°C/60 mmol/L sodium-potassium phosphate buffer, was incubated at pH 7.4 in 1.0-mL substrate (65 umol per H_2_O_2_) for 60 seconds. The enzymatic reaction was stopped by adding 1.0 mL of 32.4-mM ammonium molybdate and the yellow molybdate and H_2_O_2_ complex were measured against an unprocessed substance at 405 nm. Under these conditions, 1 unit of CAT dissociates 1 µmol H_2_O_2_/1 min. The results were expressed as kU/L calculated as below:

Serum CAT activity (kU/L) = (A (blank 2) - A (blank 3) / A (sample) - A (blank 1)) × 271

Blank 1 contained 1.0-mL substrate, 1.0-mL molybdate, and 0.2-mL sample.

Blank 2 contained 1.0-mL substrate, 1.0-mL molybdate, and 0.2-mL buffer.

Blank 3 contained 1.0-mL buffer, 1.0-mL molybdate, and 0.2-mL buffer.

Total Antioxidant Status Levels

Serum TAS was quantified by a novel automatic colorimetric measurement method developed by Erel. In this method, the antioxidant in the sample, i.e., the dark blue-green colored 2,2'-azino-bis (3-ethylbenzothiazoline-6-sulfonic acid) (ABTS), is a radical against the colorless reduced ABTS form. A change in absorbance at 660 nm is related to the total antioxidant level in the sample. This method determines the antioxidant effect of the sample against strong free radical reactions initiated by the hydroxyl radical product. The results are expressed as millimolar Trolox equivalent per liter [16].

Total Oxidant Status Level

Plasma TOS was quantified by the automatic colorimetric method developed by Erel [17]. In this method, oxidants found in the sample oxidize the iron ion-dianisidine complex to iron ion. The oxidative reaction is augmented by glycerol molecules that are abundant in the reaction medium. Iron ion forms a colorful complex with xylenol orange at acidic pH.

Spectrophotometrically quantifiable color intensity is correlated with the total amount of oxidant molecules found in the sample. The test is calibrated with hydrogen peroxide, and the results are expressed as micromolar hydrogen peroxide equivalent per liter (µmol H_2_O_2_ Eqv/L).

According to the Endocrine Society clinical practice guidelines, patients with serum D vitamin levels of less than 20 ng/mL were considered to have vitamin D deficiency [18,19].

Statistical analysis

A power analysis was performed using the t-test. Linear bivariate regression was used to compare two groups. Difference between intercepts statistical test indicated that at an alpha error probability of 0.05, study power of 0.85, number of groups of 2, and the critical t-value of 1.67, the minimum number of cases to be enrolled was 33 in the control group and 33 in the patient group. The total case number was determined to be at least 66.

Statistical analyses were performed using Statistical Package for the Social Sciences for Windows Version 22 (IBM Corp., Armonk, NY, USA). Variables are expressed as mean ± standard deviation or as number (n) and percentage (%). The Kolmogorov-Smirnov test was used to determine whether the numerical variables showed normal distribution. Student's t-test or one-way analysis of variance (ANOVA) was used for the normally distributed parameters, and Mann-Whitney U-test or Kruskal-Wallis test was used for the non-normally distributed parameters. Chi-square test, Student's t-test, or Mann-Whitney U-test was used as a statistical significance test. One-way ANOVA test was used to determine the arithmetic mean of a dependent variable between two independent groups and whether there was a significant difference. Logistic regression analysis was performed to show the relationship between one dependent variable and one or more independent variables. Correlation analysis was conducted to show the direction and severity of the relationship between two numerical variables. If the data were distributed normally, Pearson’s correlation coefficient was preferred, and if not normally, then Spearman’s rank correlation coefficient was preferred. A p-value of less than 0.05 was considered statistically significant.

## Results

The mean age of the patients was 10.74±3.38 years in the control group, 11.68±3.96 years in the NAS group, and 11.75±3.19 years in the NASH group. There was no significant difference between the study groups with respect to mean age (p = 0.413). An analysis of the sex distribution across the study groups showed that the female/male ratio was 14/21 in the control group, 36/36 in the NAS group, and 6/22 in the NASH group, indicating a statistically significant male preponderance in all groups (p = 0.038) (Table 1).

**Table 1:** Evaluation of clinical and laboratory features of the patients by groups *One-way analysis of variance: posthoc = Scheffe alpha test. **Crosstabs: chi-square test. Note: data are presented as mean ± SD for age and as N (%) for other data. NAS, nonalcoholic hepatosteatosis; NASH, nonalcoholic steatohepatitis

	Control (35)	NAS (73)	NASH (28)	p-Value
Age, years	10.74±3.38	11.68±3.96	11.75±3.19	0.413*
Gender				
Female	14 (40)	36 (49.3)	6 (21.4)	0.038**
Male	21 (60)	37 (50.7)	22 (78.6)	
Normal weight	34 (97.1)	33 (45.2)	4 (14.3)	<0.001**
Over-weight	1 (2.9)	10 (13.7)	1 (3.6)	
Obesity class 1	0 (0)	29 (39.7)	18 (64.3)	
Obesity class 2	0 (0)	1 (1.4)	5 (17.9)	
Obesity class 3	0 (0)	0 (0)	0 (0)	
Vitamin D deficiency	5 (14.3)	42 (57.5)	18 (64.3)	<0.001**
Hypercholesterolemia	0 (0)	3 (4.1)	2 (7.1)	0.313**
Hypertriglyceridemia	0 (0)	6 (8.2)	6 (21.4)	0.011**
Steatosis				
Grade 0	35 (100)	0 (0)	0 (0)	<0.001**
Grade 1	0 (0)	54 (74)	8 (28.6)	
Grade 2	0 (0)	15 (20.5)	17 (60.7)	
Grade 3	0 (0)	4 (5.5)	3 (10.7)	

Comparison based on the anthropometric measurements revealed that the NAS and NASH groups had significantly higher weight, height, BMI, and BMI Z score than the control group (p = 0.001, p < 0.001, p < 0.001, and p < 0.001, respectively) (Table 2). The rate of obesity was significantly higher in the NAS and NASH groups (p < 0.001) (Table 1).

**Table 2:** Evaluation of anthropometric measurements and laboratory findings of the patients by groups Statistics: one-way analysis of variance: posthoc = Scheffe alpha test. *Independent Student’s t-test. It shows the statistical significance between NAS and NASH. **It shows the statistical importance in one-way analysis of variance: posthoc = Scheffe test. Note: Data are presented as mean ± SD. NAS, nonalcoholic hepatosetatosis; NASH, nonalcoholic steatohepatitis; BMI, body mass index; AST, aspartate aminotransferase; ALT, alanine aminotransferase; GGT, gamma-glutamyl transferase; ALP, alkaline phosphatase; T.bil, total bilirubin; D.bil, direct bilirubin; PTH, parathyroid hormone; Ca, calcium; P, phosphor; Mg, magnesium; TAS, total antioxidant level; TOS, total oxidant status

	Control	NAS	NASH	p-Value*	p-Value**
Height Z score	-0.36±0.78	0.32±1.45	0.90±1.35	0.072	0.001
Weight Z score	-0.01±0.59	1.55±1.20	2.30±1.32	0.007	<0.001
BMI	18.49±4.04	24.90±4.12	27.96±5.61	0.012	<0.001
BMI Z score	0.58±0.92	1.59±1.02	2.15±1.18	0.022	<0.001
Glucose (mg/dL)	83±7.74	92.71±9.93	92.50±20.89	0.959	0.001
AST (U/L)	31.05±6.00	23.40±9.78	58.42±29.89	<0.001	<0.001
ALT (U/L)	32.65±4.41	20.55±10.06	99.00±67.57	<0.001	<0.001
GGT (U/L)	24.71±11.12	19.46±13.23	35.28±14.24	<0.001	<0.001
ALP (U/L)	198.11±73.66	208.54±96.69	253.19±111.41	0.058	0.062
T.bil (mg/dL)	0.44±0.18	0.49±0.31	0.51±0.34	0.784	0.592
D.bil (mg/dL)	0.15±0.06	0.34±1.35	0.21±0.19	0.620	0.614
Cholesterol (mg/dL)	154.63±19.84	151±30.61	163.93±34.25	0.087	0.140
Triglycerides (mg/dL)	77.85±14.06	114.83±59.07	150.46±67.92	0.011	<0.001
Vitamin D (ng/ml)	29.70±7.51	19.72±10.20	15.84±6.83	0.031	<0.001
PTH (pg/mL)	43.28±10.87	51.15±16.51	46.98±18.58	0.276	0.050
Ca (mg/dL)	9.98±0.33	10.08±0.51	10.04±0.45	0.628	0.597
P (mg/dL)	4.44±0.59	4.32±0.67	4.44±0.70	0.424	0.570
Mg (mg/dL)	2.05±0.21	2.13±0.18	2.12±0.13	0.750	0.117
TAS (mmol Trolox equivalent/L)	1.25±0.14	1.53±0.28	1.63±0.26	0.116	<0.001
TOS (µmol H_2_O_2_ Eqv/L)	12.67±8.06	38.74±16.10	36.01±16.90	0.706	<0.001
Catalase (kU/L)	128.13±113.06	453.04±233.95	403.97±218.79	0.339	<0.001

The rate of hypercholesterolemia was statistically similar between the study groups (p = 0.313). The rates of hypertriglyceridemia and vitamin D deficiency were significantly higher in the NAS and NASH groups compared to the controls (p = 0.011 and p < 0.001, respectively). Comparison based on hepatosteatosis grade indicated that the latter was significantly more advanced in the NASH group (p < 0.001) (Table 1).

A statistical analysis of the biochemical findings in the patient group showed that cholesterol, ALP, Ca, Mg, P, T.bil, and D.bil levels were comparable between the study groups (p = 0.140, p = 0.062, p = 0.597, p = 0.117, p = 0.570, and p = 0.592, p = 0.614, respectively). Vitamin D level was significantly lower in the NAS and NASH groups than in the control group (p < 0.001) (Table 2).

Although parathyroid hormone levels were higher in the NAS and NASH groups compared to the control group, these differences did not reach statistical significance (p = 0.050). TG and glucose levels were significantly higher in the NAS and NASH groups (p<0.001 and p = 0.001, respectively). As expected, AST, ALT, and GGT levels were significantly higher in the NASH group compared to the other groups (p < 0.001) (Table 2).

A comparison of the patients based on oxidant and antioxidant levels revealed that TAS, TOS, and CAT levels were significantly higher in the NAS and NASH groups compared with the controls (p < 0.001, p < 0.001, and p < 0.001, respectively) (Table 2).

We found significantly higher BMI, BMI Z score, and weight Z score in patients with NASH than those with NAS (p = 0.012, p = 0.022, and p = 0.007, respectively). Furthermore, patients with NASH had a significantly higher TG level (p = 0.011) and a significantly lower vitamin D level (p = 0.031) than patients with NAS (Table 2).

A comparison of the obese and non-obese NAFL patients by antioxidant levels and laboratory parameters showed that obese and non-obese patients had significantly higher glucose, TG, TAS, TOS, and CAT levels compared to the control group (p < 0.001). An analysis of the patients by AST, ALT, and GGT levels revealed that AST and ALT levels were significantly higher in the NAFL group compared to the control group, whereas GGT levels were comparable (p = 0.036, p = 0.030, and p = 0.195, respectively). We found a significantly lower vitamin D level in the NAFL group compared to the controls (p < 0.001). Parathyroid hormone levels were significantly higher in the NAFL group than in the control group (p = 0.040). A comparison of obese and non-obese NAFL patients showed that AST and ALT were significantly higher in the obese subgroup (p = 0.040 and p = 0.055, respectively), but there was no significant difference between the two subgroups with regard to glucose, vitamin D, TG, TAS, TOS, and CAT levels (p > 0.05) (Table 3).

**Table 3:** Evaluation of antioxidant levels and laboratory findings according to the control group in obese and non-obese NAFLD patients Statistics: one-way analysis of variance: posthoc = Scheffe alpha test. *Independent Student’s t-test. It shows the statistical significance between non-obese NAFLD and obese NAFLD. **It shows the statistical importance in one-way analysis of variance: posthoc = Scheffe test. Note: Data are presented as mean ± SD NAFLD, nonalcoholic fatty liver disease; AST, aspartate aminotransferase; ALT, alanine aminotransferase; GGT, gamma-glutamyl transferase; PTH, parathyroid hormone; TAS, total antioxidant level; TOS, total oxidant status

	Control	Non-Obese NAFLD	Obese NAFLD	p-Value*	p-Value**
Glucose (mg/dL)	83±7.74	91.20±9.06	93.96±16.89	0.544	<0.001
AST (U/L)	31.05±6.00	27.20±19.51	37.76±25.58	0.040	0.036
ALT (U/L)	32.65±4.41	30.39±47.75	51.63±50.64	0.055	0.030
GGT (U/L)	24.71±11.12	21.23±14.71	26.51±15.47	0.199	0.195
Vitamin D (ng/mL)	29.70±7.51	20.83±10.44	16.69±8.22	0.071	<0.001
PTH (pg/mL)	43.28±10.87	47.69±16.86	52.01±17.25	0.393	0.040
Triglycerides (mg/dL)	77.85±14.06	117.77±59.49	131±66.6	0.486	<0.001
TAS (mmol Trolox equivalent/L)	1.26±0.15	1.57±0.30	1.55±0.26	0.957	<0.001
TOS (µmol H_2_O_2_ Eqv/L)	12.67±8.06	38.05±17.78	37.92±14.98	0.999	<0.001
Catalase (kU/L)	128.13±113.06	425.49±239.01	452.06±222.70	0.813	<0.001

An analysis of the correlations between the levels of antioxidants and vitamin D, and the anthropometric measurements, clinical characteristics, and laboratory findings revealed a significant correlation between weight Z score, and TAS, TOS, and CAT levels (r: 0.282, p = 0.001; r: 0.378, p < 0.001; and r: 0.317, p < 0.001, respectively). Weight Z score had a significant negative correlation with vitamin D level (r: -0.423; p < 0.001). There was a weak positive correlation between height Z score and TAS and TOS (r: 0.186, p = 0.036 and r: 0.196, p = 0.022, respectively) and a weak negative correlation with vitamin D (r: -0.179 p = 0.038). Significant positive correlations were detected between body mass index Z score, and TAS, TOS, and CAT levels (r: 0.268, p = 0.002; r: 0.386, p < 0.001; and r: 0.352, p < 0.001, respectively). There was a significant negative correlation between vitamin D level and BMI Z score (r: -0.432; p < 0.001). There was no correlation between cholesterol level, and TAS, TOS, CAT, and vitamin D levels. There was a weak positive correlation between TG level, and TAS and CAT levels (r: 0.217, p = 0.011 and r: 0.181, p = 0.035, respectively), and a negative correlation was found with vitamin D level (r: -0.295; p = 0.001) (Table 4).

**Table 4:** Evaluation of the correlation of antioxidant and vitamin D levels with clinical and laboratory findings Statistics: Pearson’s correlation *Correlation is significant at the 0.01 level (two-tailed). **Correlation is significant at the 0.05 level (two-tailed). TAS, total antioxidant level; TOS, total oxidant status; BMI, body mass index

		TAS	TOS	Catalase	Vitamin D
Weight Z score	r	0.282^*^	0.378^*^	0.317^*^	-0.423^*^
	p-value	0.001	<0.001	<0.001	<0.001
Height Z score	r	0.186^**^	0.196*^*^	0.119	-0.179^**^
	p-value	0.036	0.022	0.166	0.038
BMI Z score	r	0.268^*^	0.386^*^	0.352^*^	-0.432^*^
	p-value	0.002	<0.001	<0.001	<0.001
Cholesterol	r	0.078	0.021	0.016	-0.060
	p-value	0.368	0.805	0.855	0.487
Triglycerides	r	0.217^*^	0.128	0.181^**^	-0.295^*^
	p-value	0.011	0.137	0.035	0.001
Hepatosteatosis Grade	r	0.330^*^	0.439^*^	0.342^*^	-0.473^*^
	p-value	<0.001	<0.001	<0.001	<0.001
Vitamin D	r	-0.163	-0.375*	-0.298*	1
	p-value	0.059	<0.001	<0.001	

Positive correlations were found between hepatosteatosis grade, and TAS, TOS, and CAT levels (r: 0.330, p < 0.001; r: 0.439, p < 0.001; and r: 0.342, p < 0.001, respectively). We also found a negative correlation between vitamin D level and hepatosteatosis grade (r: -0.473; p < 0.001) (Table 4).

## Discussion

As far as we know, this study is the first to assess the relationship between the levels of oxidant-antioxidants and vitamin D, and other clinical and laboratory parameters in patients with NAFL. This study revealed that vitamin D was significantly lower in the NAS and NASH groups; it also showed a negative correlation with some clinical and biochemical parameters. It also demonstrated that the oxidant-antioxidant levels were significantly higher in the NAS and NASH groups and showed a positive correlation with some clinical and biochemical profiles.

It has been previously stressed that obesity, as well as body fat accumulation that induces reactive oxygen radical formation independently of obesity, may be the actual source of reactive oxygen radicals that are responsible for oxidative stress in obese individuals, giving rise to obesity-related complications such as insulin resistance, diabetes, hypertension, and atherosclerosis [20,21].

Sfar et al. [22] reported that obesity-induced increase in oxidant stress may also be seen in the childhood period. They also emphasized that obesity may be an independent risk factor for free radical generation and may augment the antioxidant response [22]. Nearly half of our NAFL patients comprised obese individuals. We demonstrated significantly higher TAS, TOS, and CAT levels in obese and non-obese NAFL patients compared with the control group (p < 0.001). There was, however, no significant difference between the obese and non-obese patients with NAFL with respect to antioxidant levels (p > 0.05) (Table 4). However, there was a positive correlation between weight Z score, BMI Z score, and antioxidant levels. We are of the opinion that the higher antioxidant levels in non-obese NAFL patients result from fatty liver triggering the formation of reactive oxygen radicals independently of obesity. Likewise, Dursun et al. [14] reported no significant difference between the vitamin D levels of obese patients with and without hepatosteatosis and stressed that obesity was correlated with vitamin D levels independently of NAFL. Our study differs from previous studies by showing that vitamin D levels were significantly lower in patients with NASH than those with NAS. This difference suggests that vitamin D may play a role in the pathogenesis of NASH among patients with NAFL.

Vitamin D is classically known as a hormone with important functions for maintaining mineral metabolism and musculoskeletal health. It also possesses antioxidant properties that are as potent as or more potent than those of the classical antioxidant vitamin E [23,24]. Furthermore, it has been discovered that vitamin D is a potent hormone with important biological functions such as induction of cellular differentiation, alleviation of inflammation, and immunomodulation [25]. Lee et al. reported that vitamin D increased antioxidant enzyme levels and suppressed lipid peroxidation, thereby mitigating oxidative stress [24]. Another study [26] indicated that vitamin D deficiency was related to hepatosteatosis, and children with vitamin D deficiency were at a greater risk of having illness due to oxidative stress. We demonstrated that vitamin D level was reduced as the TG level, hepatosteatosis stage, weight, and BMI Z score increased. These findings support the notion that vitamin D deficiency, obesity, and hepatosteatosis induce oxidative stress, which has already been reported in the literature.

Besagil et al. [27] reported no significant difference regarding serum total antioxidant capacity (TAC) levels between obese and normoweight patients. They suggested that this may have stemmed from other factors such as the degree and duration of obesity, nutritional habits, and current diet. Furthermore, they showed a positive correlation of TAS levels with weight and BMI. We also noted higher TAS, TOS, and CAT levels in NAFL patients compared to the control group. Moreover, we showed a positive correlation between the hepatosteatosis grade, weight, and BMI Z score, and TAS, TOS, and CAT levels (Table 4). We believe that increased antioxidant levels are likely a response to increased levels of free oxygen radicals due to oxidative stress. Albuali [28] demonstrated that children who had BMI > 35 kg/m^2^ had higher levels of oxidative stress products and lower levels of antioxidant molecules compared to those with BMI < 35 kg/m^2^. Our study enrolled four patients with a BMI greater than 35 kg/m^2^. Those patients had no significantly different TAS, TOS, and CAT levels compared to NAFL patients with lower BMI levels (p = 0.413, p = 0.093, and p = 0.193, respectively). This may have resulted from a lower number of patients with BMI > 35 kg/m^2^.

A study on individuals with coronary heart disease indicated that those with coronary heart disease had higher body fat percentage, waist circumference, BMI, serum TG level, and plasma TAC concentration compared to the healthy controls [29]. It is known that in patients with type 2 diabetes, chronic hyperglycemia is linked to increased lipid peroxidation secondary to oxidative stress [24]. Our study supports the literature data in that it found significant increases in glucose, TG, TAS, TOS, and CAT levels, as well as BMI Z score in NAFL patients compared to the controls. Kilic et al., on the other hand, demonstrated higher TAS and TOS levels in obese children than normoweight children. They reported that obesity caused an increase in TOS levels, with TAS levels having increased to counterbalance it [30]. We demonstrated that obese and non-obese NAFL patients had significantly higher TAS, TOS, and CAT levels compared to the control group (p < 0.001). However, no significant difference existed between obese and non-obese patients within the NAFL group (Table 3). This suggests that not only obesity but also fatty liver increases oxidative stress products (TOS). In response to this situation, antioxidant levels may also increase.

The limitations of our study include the lack of addressing environmental factors such as patients’ dietary components, activities, daily caloric intakes, daily dietary antioxidant intakes, household smoking statuses, and daily sun exposures. However, as the only study investigating the relationship between oxidative stress, antioxidant levels, vitamin D, and other biochemical parameters, our study makes a valuable contribution to the literature. Furthermore, our study will shed light on future studies that would assess the role of vitamin D and antioxidant treatment in patients with NAFL.

## Conclusions

In conclusion, our study demonstrated that vitamin D level was significantly lower and the levels of oxidant-antioxidants including TOS, TAS, and CAT were higher among patients with NAFL. More studies are needed to understand the importance of vitamin D and oxidant-antioxidant substances in the pathogenesis and treatment of NAFLD.
